# Functional analysis of androgen receptor mutations that confer anti-androgen resistance identified in circulating cell-free DNA from prostate cancer patients

**DOI:** 10.1186/s13059-015-0864-1

**Published:** 2016-01-26

**Authors:** Nada Lallous, Stanislav V. Volik, Shannon Awrey, Eric Leblanc, Ronnie Tse, Josef Murillo, Kriti Singh, Arun A. Azad, Alexander W. Wyatt, Stephane LeBihan, Kim N. Chi, Martin E. Gleave, Paul S. Rennie, Colin C. Collins, Artem Cherkasov

**Affiliations:** Vancouver Prostate Centre, University of British Columbia, 2660 Oak St., Vancouver, BC V6H 3Z6 Canada; Department of Medical Oncology, BC Cancer Agency, 600 West 10th Avenue, Vancouver, BC V5Z 4E6 Canada; Laboratory for Advanced Genome Analysis (LAGA), Vancouver Prostate Centre, 2660 Oak St., Vancouver, BC V6H 3Z6 Canada

**Keywords:** Androgen receptor, Castration-resistant prostate cancer, Cell-free circulating DNA, Mutations, Drug resistance, Anti-androgens and steroids

## Abstract

**Background:**

The androgen receptor (AR) is a pivotal drug target for the treatment of prostate cancer, including its lethal castration-resistant (CRPC) form. All current non-steroidal AR antagonists, such as hydroxyflutamide, bicalutamide, and enzalutamide, target the androgen binding site of the receptor, competing with endogenous androgenic steroids. Several AR mutations in this binding site have been associated with poor prognosis and resistance to conventional prostate cancer drugs. In order to develop an effective CRPC therapy, it is crucial to understand the effects of these mutations on the functionality of the AR and its ability to interact with endogenous steroids and conventional AR inhibitors.

**Results:**

We previously utilized circulating cell-free DNA (cfDNA) sequencing technology to examine the AR gene for the presence of mutations in CRPC patients. By modifying our sequencing and data analysis approaches, we identify four additional single AR mutations and five mutation combinations associated with CRPC. Importantly, we conduct experimental functionalization of all the AR mutations identified by the current and previous cfDNA sequencing to reveal novel gain-of-function scenarios. Finally, we evaluate the effect of a novel class of AR inhibitors targeting the binding function 3 (BF3) site on the activity of CRPC-associated AR mutants.

**Conclusions:**

This work demonstrates the feasibility of a prognostic and/or diagnostic platform combining the direct identification of AR mutants from patients’ serum, and the functional characterization of these mutants in order to provide personalized recommendations regarding the best future therapy.

**Electronic supplementary material:**

The online version of this article (doi:10.1186/s13059-015-0864-1) contains supplementary material, which is available to authorized users.

## Background

Advances in prostate cancer (PCa) research have led to the development of novel therapies for the metastatic castration-resistance (CRPC) form of the disease, such as two recent drugs abiraterone [1, 2] and enzalutamide [3, 4], which target the androgen receptor (AR) pathway. Abiraterone inhibits cytochrome P450 17A1 (CYP17A1), an enzyme responsible for the synthesis of testosterone that, after conversion to dihydrotestosterone (DHT), binds to the androgen binding site (ABS) of the AR and activates the AR signaling axis. Enzalutamide is a potent anti-androgen that competes with DHT and binds to the ABS, preventing AR transcriptional activation. Unfortunately, patients with advanced PCa either do not respond to anti-androgen therapy due to pre-existing aberrations of CYP17, or AR or relapse to CRPC due to adaptive responses, or Darwinian selection of rare aberrations. Thus, while these therapies improve disease management and extend life for most patients, ultimately they are only palliative.

In the majority of cases, CRPC is accompanied by reactivation of the AR signaling axis so that the receptor regulates its numerous target genes including PSA. Not surprisingly, an impressive repertoire of mechanisms has been identified that reactivate the AR signaling axis. These include upregulation of CYP17 [5], amplification of the AR gene [6], expression of constitutive AR splice variants [7, 8], or mutation of the AR itself [9–12]. It has been demonstrated that mutations in the ABS of the AR can lead to its activation by weak adrenal androgens, steroidal and non-steroidal ligands, and by mutation-driven conversion of AR inhibitors into agonists [13]. For example, the AR substitution T878A, identified in the LNCaP cell line, confers resistance to the anti-androgen hydroxyflutamide [14] and is promiscuously activated by progesterone and 17β-estradiol [15]. Other mutations such as W742C/L and F877L are associated with resistance to the anti-androgens bicalutamide [16–18] and enzalutamide [19–21], respectively. Thus, identification and characterization of resistance-associated AR mutations, as biomarkers for primary treatment of both naïve PCa and CRPC patients, are critically important for predicting, as well as monitoring, patient’s response to therapy. This process is essential for the development of evidence-based precision oncology.

The detection of circulating cell-free DNA (cfDNA) has recently emerged as a non-invasive diagnostic tool for a variety of cancers, including CRPC, and as a technology maximizing the efficacy of anticancer therapies [22, 23]. It is estimated that up to 3 % of tumor DNA is released into the circulatory system daily from the processes such as secretion, necrosis, and primarily apoptosis [24]. In our previous work [25], we reported the development of a sequencing platform that allowed detection of a repertoire of AR mutations in cfDNA isolated from CRPC patients. This method enabled effective sequencing of AR exon 8 from plasma samples of 47/62 metastatic CRPC patients who were progressing on systemic therapy, and thus resulted in the identification of numerous AR mutations, including three previously unreported ones.

By improving the sequencing and data analysis processes, we were able to sequence the cfDNA of the 15 CRPC patients that were previously excluded due to low yield of DNA in their plasma samples, to validate all of the mutations reported in our previous work [25] and to identify new candidate mutations.

**Fig. 1:**
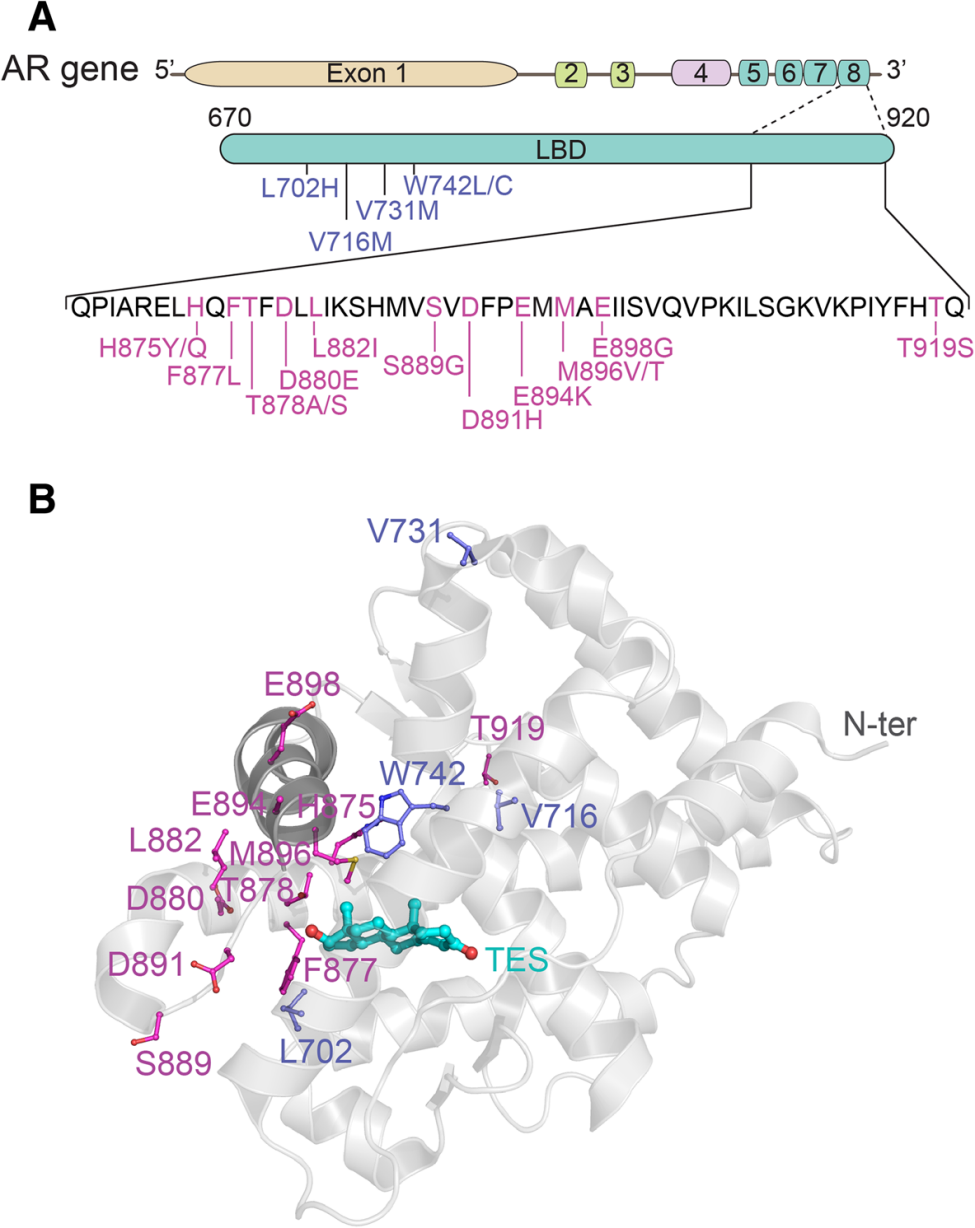
AR mutations identified in CRPC patients. **a** AR gene organization showing the AR-LBD mutants. **b** AR mutants mapped on the X-ray structure (PDB: 2 AM9) of the LBD (cartoon representation, in gray) in complex with testosterone (TES, ball-and-stick representation, in cyan). AR mutants encoded by exon 8 are shown in magenta ball-and-stick representation. The rest of the mutants are shown in blue

Importantly, in the current work, we have also carried out *in vitro* characterization of all AR mutations identified in 62 CRPC patients together with seven AR mutants previously reported in the literature (L702H, W742L, W742C, V716M, V731M, T878S, and M896T), to ascertain the exact mechanisms of resistance to AR pathway inhibitors (Fig. 1). To accomplish this task, we engineered each one of 24 distinct AR mutants (containing single and multiple amino-acid substitutions), and determined *in vitro* effects of four current AR antagonists (enzalutamide, hydroxyflutamide, bicalutamide, and ARN509) on all mutants, as well as investigated their *in vitro* responses to four different steroids including DHT, progesterone, estradiol, and hydrocortisone. As the result, we present evidence that all identified AR mutations provide evolutionary escape routes from androgen blockade, thus highlighting the need for novel AR inhibitors that bind to the AR outside of the ABS. Finally, we demonstrate that VPC-13566, one of our recently developed class of AR inhibitors bearing a quinolone scaffold [26] that directly interferes with AR recruitment of co-chaperones and activating cofactors via binding to the BF3 surface [27, 28], effectively inactivates the AR signaling axis for all 24 CRPC-associated AR mutants.

## Results

### Deep sequencing reveals AR mutations in cfDNA

In the current study, we used data from a patient cohort we previously reported [25]. We showed that mutations in the AR ABS contributed to treatment resistance in a subset of patients and presented the possibility of detecting these mutations in cfDNA at the point of progression [25]. Due to low DNA yield (<30 ng), 15 patients were not amenable to sequencing. In order to overcome this limitation, we have WGA2-amplified and sequenced cfDNA from these patients and modified the pipeline we developed previously [25] to enable detection of mutations in WGA2 cfDNA (see the ‘Methods’ section for more details). We have also performed experimental validation of the redesigned pipeline using direct comparison of WGA2 and non-amplified data for subset of cfDNA samples as well as alternative sequencing platforms (see Additional file 1: Supplementary data, Table S1).

In total, mutations were detected at 13 nucleotide positions in the coding region of exon 8 in 14/62 (23 %) of patients (Table 1). The frequency of these mutations in patients’ cfDNA ranged from 0.11 % to 23 %. Mutations at two positions were silent, while mutations in the remaining 11 resulted in 12 distinct amino-acid substitutions (no nonsense mutations were detected). Two missense mutations were detected in multiple patients: H875Y (n = 7) and T878A (n = 4). By including the WGA2 sequencing, we were able to report four new mutations (H875Q, D891H, E898G, and T919S) that were neither identified in our previous study [25] nor described in the literature.

**Table 1:** AR mutations detected in CRPC patients

Patient	Amino acid change	Mutant read count	Wild-type read count	Total read count	Percent mutant
VC-001-t1*	T878A	39	13,231	13,270	0.29
	H875Y	36	13,351	13,387	0.27
	H875Y/T878A	251	13,019	13,270	1.89
VC-001-t2	T878A	30	12,626	12,656	0.24
	H875Y	0	13,307	13,307	0.00
	H875Y/T878A	230	12,426	12,656	1.82
	T878A/D891H	158	10,383	10,541	1.50
	D891H	8	10,533	10,541	0.08
VC-005	E894K	170	10,745	10,915	1.56
VC-012-t1	M896V	1,270	5,985	7,255	17.51
VC-012-t1*	S889G	307	4,769	5,076	6.05
	M896V	273	5,838	6,111	4.47
VC-012-t2	S889G	103	8,202	8,305	1.24
	M896V	31	8,934	8,965	0.35
	H875Y	49	10,355	10,404	0.47
	T878A	300	9,760	10,060	2.98
	F877L/T878A	141	9,919	10,060	1.40
	T878A/S889G	35	8,270	8,305	0.42
VC-014*	E898G	237	11,919	12,156	1.95
VC-015	T878A	218	9,502	9,720	2.24
VC-017	T878A	99	12,626	12,725	0.78
VC-018*	H875Y	223	10,382	10,605	2.10
VC-021*	H875Q	251	9,846	10,097	2.49
	T919S	238	9,004	9,242	2.58
VC-022	D880E	12	10,902	10,914	0.11
VC-040	H875Y	479	7,560	8,039	5.96
VC-041-t1	H875Y	1,521	7,260	8,781	17.32
VC-041-t2	H875Y	4,665	15,662	20,327	22.95
VC-053	H875Y	136	9,064	9,200	1.48
VC-063	H875Y	270	14,874	15,144	1.78
VC-064	L882I	17	15,670	15,687	0.11

Newly reported samples sequenced from WGA2 DNA are marked with a *. Each horizontal line in the table represents a particular haplotype, hence multiple lines for some data points. Only patients with mutations detected in cfDNA or in WGA2 cfDNA are shown

**Fig. 2:**
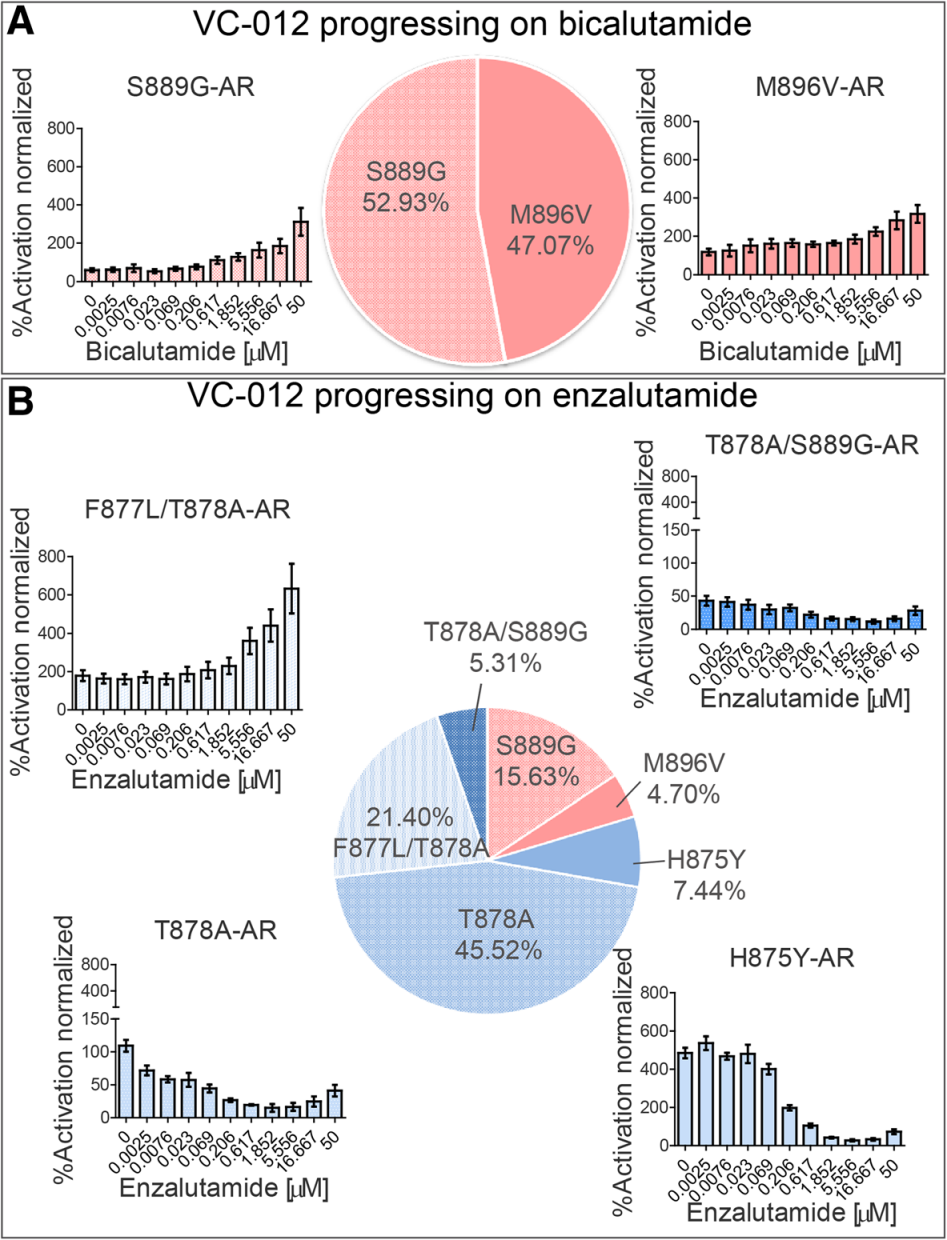
Characterization of AR mutants identified in patient VC-012 after progression on bicalutamide and enzalutamide. **a** Two AR mutants were identified in the cfDNA isolated after patient progression on bicalutamide. Both mutants show agonist response to bicalutamide in an *in vitro* transcription assay. **b** Four additional mutants were identified in the same patient VC-012 after progression on enzalutamide, all with various agonist effects toward enzalutamide *in vitro*. The percentage in the charts only reflects the mutated form of the androgen receptor. Each concentration was assayed in quadruplicate n = 4, with a biological replicate of n = 3. Results were averaged and normalized by expressing them as a percentage of the wild-type AR activity ± SEM

We previously discussed the validation of sequencing results using MiSeq resequencing of AR exon 8 amplicons and additional DNA samples from VC-012 and VC-041 patients. Inclusion of WGA2 sequencing data allowed us to extend the validation. For example, we have identified M896V and S889G mutations in the WGA2 sequence of the patient VC-012 at the first time-point; both mutants were supported by the unamplified sequence data from the first and/or the second time-points. We reported that for VC-012, four additional mutations were identified at the second (post-enzalutamide) time-point, including F877L/T878A and T878A/S889G (Fig. 2).

The cfDNA sample from the first time-point of VC-001 patient was collected at progression on abiraterone prior to commencing enzalutamide, but the patient’s mutation status was not reported due to low cfDNA yield. The second sample was collected approximately 3.5 months later at the point of progression on Enzalutamide. After WGA2 sequencing, two single (H875Y and T878A) and one combined (H875Y/T878A) mutation were detected at the first time-point, in addition to a silent mutation L874L (Table 1). Two additional substitutions, D891H and T878A/D891H, were detected at enzalutamide progression. Similar to patient VC-012, we detected multiple AR haplotypes at both time-points for VC-001, none of which contained more than two missense mutations.

### AR transcriptional activation by steroids

**Fig. 3:**
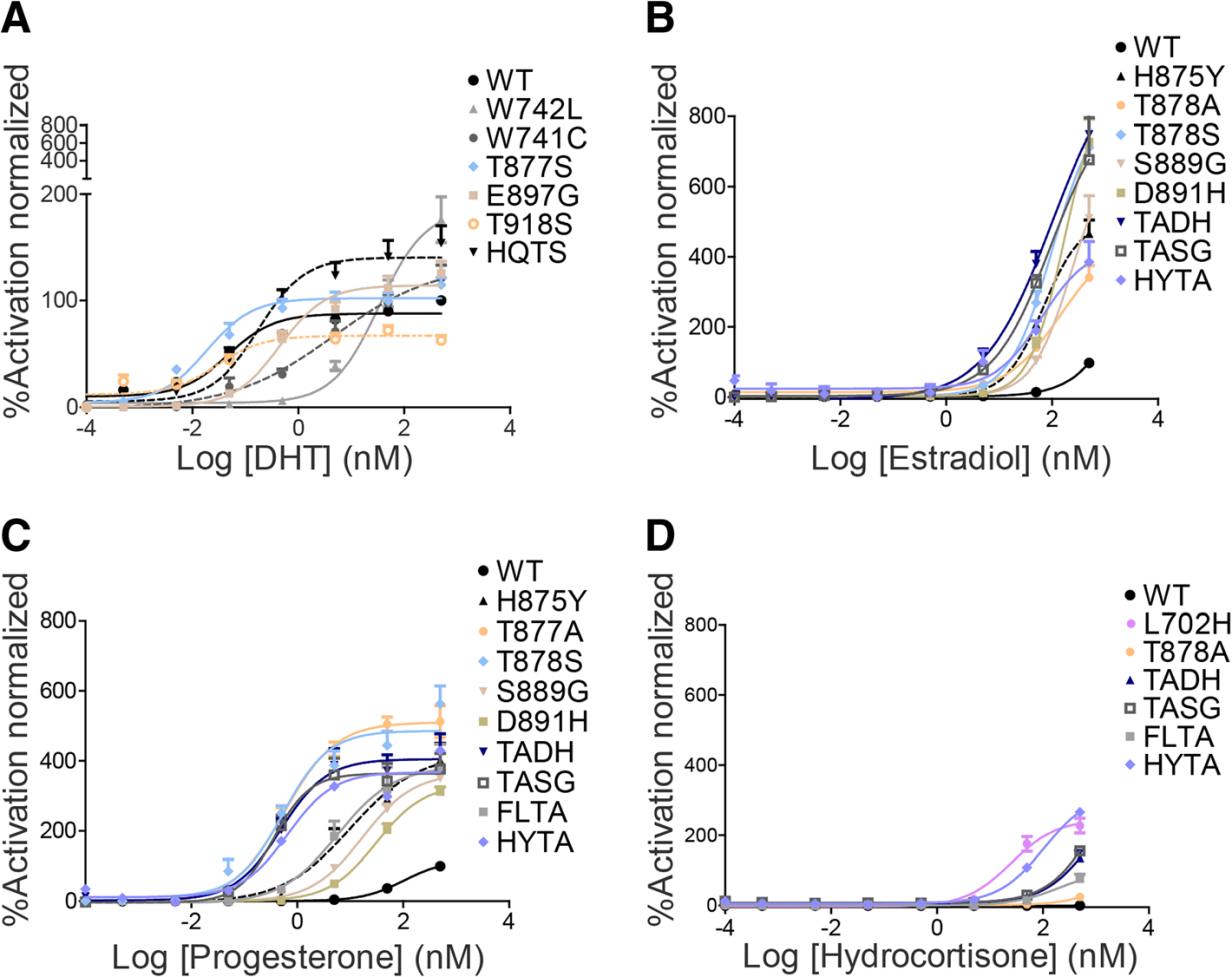
Steroid activation of AR mutants in comparison with the wild-type receptor in luciferase reporter assay. While most of the AR mutants showed similar or lower affinity to the activation by DHT (**a**), when compared to wild-type, several variants presented better activation by estradiol (**b**), progesterone (**c**), or hydrocortisone (**d**) than the wild-type. PC3 cells were transfected with both wild-type or mutated AR and a reporter plasmid pARR3-tk-luciferase. After 48 h post transfection, cells were treated with increasing concentrations of steroids. The graphs represent the average ± SEM of three independent experiments with four replicates each. The activity of each mutant in the presence of a steroid was normalized to the wild-type stimulated by 500 nM of the same steroid

The response of AR mutants to increasing concentrations of DHT has been measured using a luciferase-reporter transcription assay in PC3 cells transiently transfected with either wild-type or mutated AR. The expression level of all of the mutants was evaluated by western blotting (Additional file 2: Figure S1). Only two point mutations, T878S (EC50 = 0.019 nM) and T919S (EC50 = 0.030 nM), made the receptor slightly more sensitive to DHT compared to the wild type (EC50 = 0.047 nM) (Table 2). Some mutants such as H875Q/T919S and W742L/C appeared to be over-stimulated by higher concentrations of DHT (Fig. 3a). A noteworthy mutant was W742L that showed an approximately two-fold higher level of transcriptional activity than the wild-type AR at high concentrations of DHT (approximately 500 nM) (Table 2, Fig. 3a).

**Table 2:** The inhibition of AR mutants by VPC-13566 and their activation by various steroids

AR construct	IC_50_ of VPC-13566 Inhibition (μM)	EC_50_ of DHT activation (nM)	EC_50_ of estradiol activation (nM)	EC_50_ of progesterone activation (nM)	EC_50_ of hydrocortisone activation (nM)
WT	1.73	0.05	>500	104.0	>500
L702H	6.13	8.00	>500	172.0	25.0
V716M	1.06	0.14	>500	329 .0	>500
V731M	0.99	0.09	>500	115.0	>500
W742L	2.29	33.60	>500	>500	>500
W742C	3.43	4.74	>500	293.0	>500
H875Y	1.34	0.14	68.0	10.20	>500
H875Q	0.79	0.43	>500	>500	>500
F877L	0.37	0.08	>500	>500	>500
T878A	2.56	0.06	144.0	0.57	>500
T878S	0.43	0.02	100.0	0.53	>500
D880E	1.14	0.11	>500	177.0	>500
L882I	0.84	0.20	>500	>500	>500
S889G	10.48	0.37	230.0	17.20	>500
D891H	2.35	0.12	173.0	31.0	>500
E894K	1.20	0.25	>500	143.0	>500
M896V	0.59	4.50	>500	>500	>500
M896T	0.10	>500	>500	>500	>500
E898G	1.24	0.45	>500	>500	>500
T919S	1.29	0.03	>500	123.0	>500
H875Q/T919S	0.63	0.18	>500	>500	>500
T878A/S889G	13.20	0.12	94.0	0.36	>500
T878A/D891H	13.40	0.40	100.0	0.49	>500
H875Y/T878A	10.80	1.26	63.0	0.66	105.0
F877L/ T878A	11.70	0.81	>500	5.70	>500

The IC50 values of the inhibition by VPC-13566 and the EC50 values of the activation by DHT, estradiol, progesterone, and hydrocortisone are reported for the wild-type AR and the 24 studied mutants. For steroid activation, we tested a concentration range up to 500 nM, therefore mutants showing no activation or very weak activation in the studied range are presented with EC50 values >500 nM

Taken together, these data illustrate the heterogeneous responses of AR mutants towards activation by DHT; ranging from mutation-driven enhancement of AR ligand sensitivity, to creation of ‘super-active’ variants of the receptor. It is noteworthy that these CRPC mutants may also present higher affinities toward other steroids in order to overcome the effect of androgen deprivation [29–31]. Therefore, we tested the response of wild-type and mutated AR to activation by three other steroids: estradiol, progesterone, and hydrocortisone. The wild-type AR was only mildly stimulated by progesterone concentrations higher than 100 nM and was not activated with estradiol or hydrocortisone at concentrations as high as 500 nM (Additional file 3: Figure S2). Regarding activation with estradiol, many mutants demonstrated a stronger response to this steroid compared to the wild-type receptor (Fig. 3b). It has been reported that H875Y, T878A, and T878S could be activated by estrogens (reviewed in [13] and validated in our assay). In the current work, we show that some of the newly reported mutants such as D891H, T878A/D891H, and T878A/S889G were stimulated up to eight-fold higher than the wild-type receptor in the presence of 500 nM estradiol (Fig. 3b, Table 2).

Nine AR mutants could be effectively stimulated by much lower concentrations of progesterone compared with the wild-type AR (EC50 = 104 nM). For example, mutant T878S revealed an EC50 value of 0.53 nM (progesterone), which is 200 times higher affinity than the wild-type AR. Similarly, mutants T878A/S889G, T878A, T878A/D891H, F877L/T878A, H875Y, H875Y/T878A, S889G, and D891H exhibited EC50 in the range of nanomolar progesterone concentrations (Fig. 3c, Table 2). Some of these mutants were previously reported to be activated by progesterone (such as T878A/S and H875Y) [9, 13, 31, 32].

The wild-type AR (and most of the mutants) did not exhibit any significant transcriptional activation with hydrocortisone at a concentration up to 500 nM, with the exception of the L702H mutant (EC50 = 25 nM) known to be activated by hydrocortisone [15] and H875Y/T878A (EC50 = 105 nM) (Fig. 3d, Table 2).

### AR transcriptional inhibition by AR antagonists

We have tested four current non-steroidal AR antagonists: hydroxyflutamide [14], bicalutamide [16, 17], enzalutamide [33–35], and ARN509 [36] (Table 3, Additional file 4: Table S2) for their effects on the transcriptional activity of 24 AR mutants. Using the same luciferase transcription assay described above, cells were stimulated with the non-metabolizable androgen R1881 and then treated with the increasing concentrations of the drugs. This experiment allowed us to identify specific AR mutations that decrease the sensitivity of the receptor to inhibition by these antagonists as well as the characterization of the mutations that transform current anti-androgens into AR agonists. Thus, in addition to the already well-documented F877L mutation that converts enzalutamide to a partial agonist, we showed that the compound mutation (F877L/T878A), that is also present in the enzalutamide resistant cell line MR49F [37], converted this drug into a full agonist. We have also observed that canonical hydroxyflutamide-resistant T878A and H875Y AR variants also confer a partial agonist effect to enzalutamide when the drug was administered at higher concentrations (Table 3, Fig. 2). When T878A is combined with other LBD mutations, such as T878A/D891H or T878A/S889G, the activation effect of enzalutamide on the mutant is retained, and seemingly enhanced. Of note, in the panel of 24 studied AR variants, the experimental drug ARN509 behaved very similarly to enzalutamide (Table 3 and Additional file 4: Table S2), which was not surprising, considering that a very high degree of structural resemblance exists between the two chemicals. Moreover, our results indicate that two mutants - H875Y and F877L - appear to be more resistant to ARN509 than to enzalutamide (Additional file 4: Table S2).

**Table 3:** The response of CRPC-associated AR mutations to anti-androgen treatments

AR mutants	Agonist response to treatment			
	Hydroxyflutamide	Bicalutamide	Enzalutamide	ARN509
L702H	Partial	Partial	No	No
V716M	Partial	Partial	No	No
V731M	Partial	Partial	No	No
W742L	Partial	Yes	No	No
W742C	Partial	Yes	No	No
H875Y	Yes	Partial	Partial	Partial
H875Q	Partial	Partial	No	No
F877L	Partial	No	Partial	partial
T878A	Partial	Yes	Partial	partial
T878S	Partial	Yes	Partial	partial
D880E	Partial	Partial	No	No
L882I	Partial	Partial	No	No
S889G	Yes	Yes	No	No
D891H	Yes	Yes	No	No
E894K	Partial	Partial	No	No
M896V	Partial	Yes	No	No
M896T	Partial	Yes	No	No
E898G	Partial	No	No	No
T919S	Partial	Partial	No	No
H875Q/T919S	Partial	Partial	No	No
T878A/S889G	Yes	Yes	Partial	Partial
T878A/D891H	Yes	Yes	Partial	No
F877L/ T878A	No	Yes	Yes	Yes
H875Y/T878A	Yes	Yes	Partial	Partial

Results presented in Additional file 4: Table S2 are summarized here. We considered as partial agonist a drug that inhibited a mutant at low concentrations and stimulated its activity at high concentrations

**Fig. 4:**
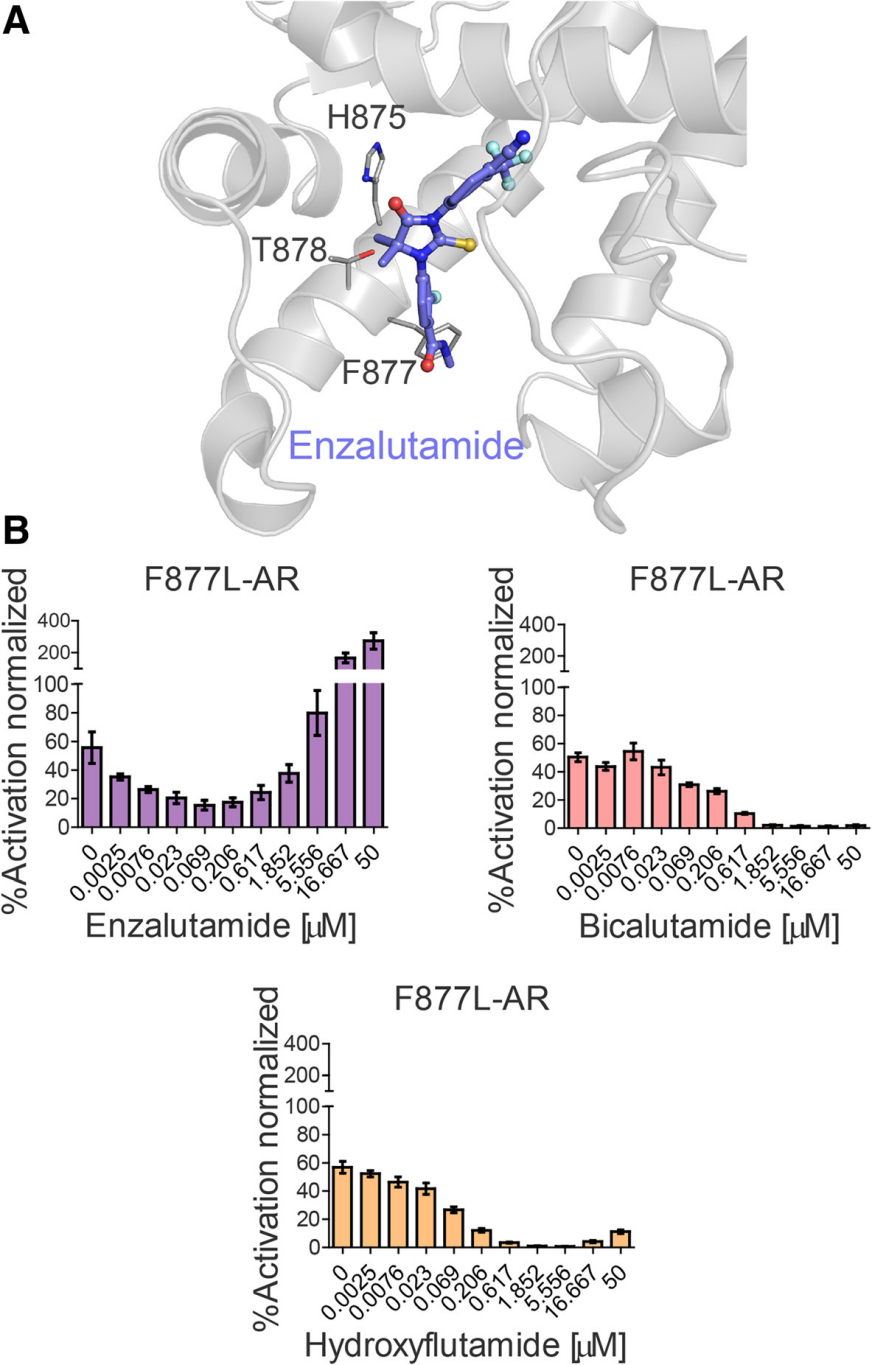
AR mutants associated with enzalutamide resistance in CRPC patients. **a** Molecular dynamics (MD) model of AR LBD (cartoon representation, in gray) in complex with enzalutamide (ball-and-stick representation, in blue). The residues presented as gray sticks are found to be mutated in patients progressing on enzalutamide treatment. **b** The F877L mutant showed an agonist response to enzalutamide in an *in vitro* cell-based assay but was inhibited by the first generation anti-androgens hydroxyflutamide and bicalutamide. Each concentration was assayed in quadruplicate n = 4, with a biological replicate of n = 3. Results were averaged and normalized by expressing them as a percentage of WT AR activity ± SEM

In accordance with a previous study [20], we found that the well-documented enzalutamide- and ARN509-resistant mutant, F877L, can effectively respond to the older anti-androgens, bicalutamide and hydroxyflutamide (Fig. 4, Additional file 4: Table S2). A similar observation was also made for the double mutant F877L/T878A that provides a profound agonist function to hydroxyflutamide, enzalutamide, and ARN509, but can be effectively suppressed by bicalutamide (Table 3, Additional file 4: Table S2). These important cases illustrate that the use of cfDNA sequencing technology could revive older treatment options for some CRPC patients that have developed resistance to enzalutamide. These results once again emphasize the importance of an evidence-based approach to precision oncology for prostate cancer patients.

We recently reported that H875Y and T878A AR mutations were identified in patients progressing on abiraterone or had previously received it [25]. Romanel *et al.* also showed the emergence of T878A and L702H mutants in 13 % of patients progressing on abiraterone [38]. As none of the tested mutants were activated with abiraterone in our assay (Additional file 4: Table S2), the selection for these mutants after abiraterone treatment seems important for the AR promiscuous activation by other steroids, especially progesterone. This suggestion could be supported by the increase in progesterone levels after abiraterone therapy [39].

### Inhibition of AR mutants via BF3 site

**Fig. 5:**
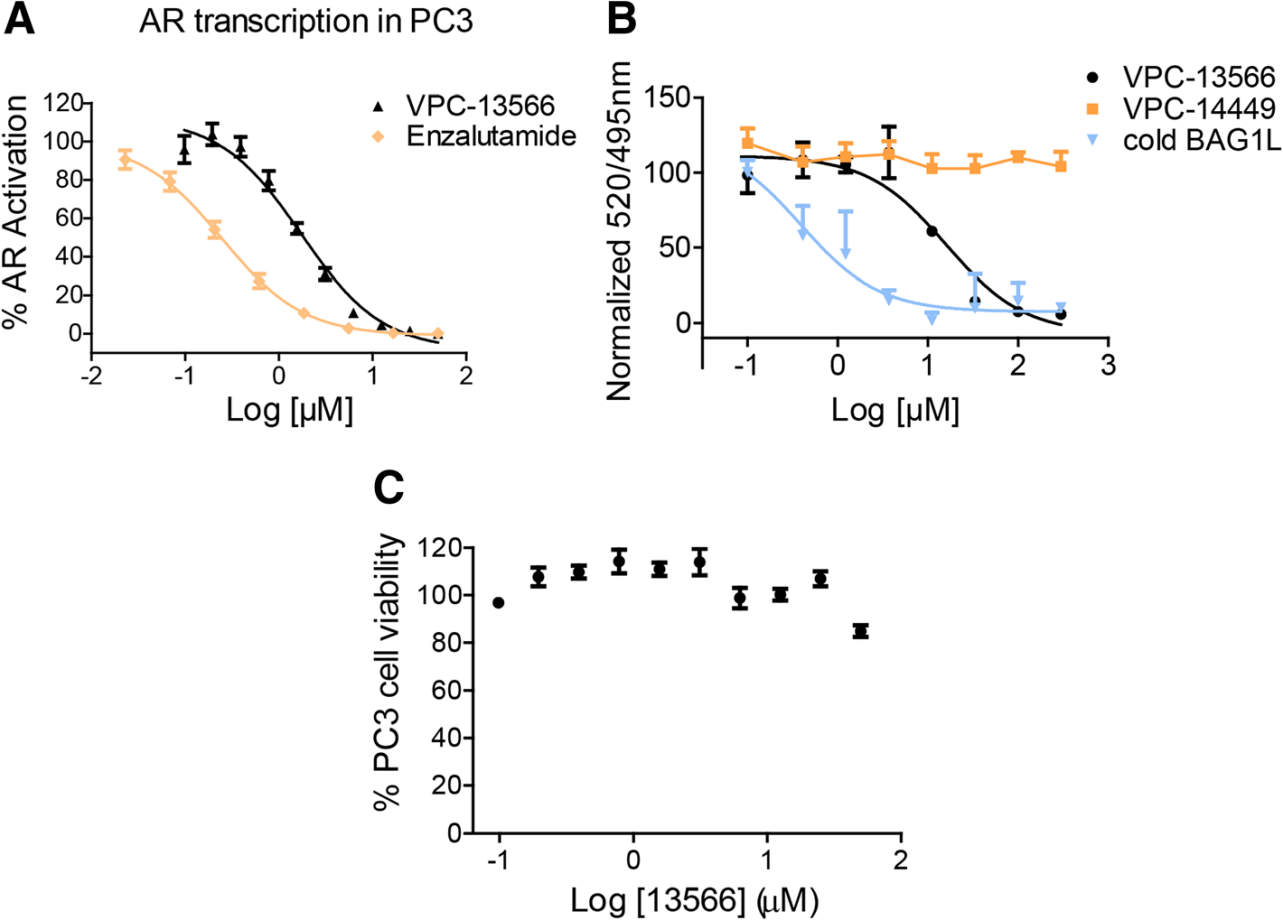
Characterization of the in-house developed AR inhibitor VPC-13566. **a** Dose-response curve illustrating the inhibiting effect of the VPC-13566 and enzalutamide on the AR transcriptional activity in PC3 cells transfected with wild-type AR plasmid. Data points represent the mean of three independent experiments performed in four replicates each. Error bars represent the standard error of the mean ± SEM for n = 12 values. **b** The specific binding to the BF3 site was confirmed by BAG1L peptide (1-20) displacement using a TR-FRET assay. **c** The effect of VPC-13566 on PC3 cell viability. % cell viability is plotted in dose dependent manner. Data points represent the mean ± SEM of two independent experiments performed in quadruplicate

One promising strategy for combating mutation-driven drug resistance could be to develop drugs that act on the AR outside of the ABS region. This paradigm is exemplified by the recently described Binding Function-3 (BF3) - a protein-protein interaction site that is essential for AR transcriptional activity and is involved in recruiting AR co-regulators such as FKBP52 and Bag-1 L [27, 40]. Previously, we reported on the development of quinolone derivatives that selectively inhibit the AR through its BF3 functionality at clinically relevant concentrations [26, 41–44]. One such AR inhibitor - 2-(7-methyl-1H-indol-3-yl) quinolone (called VPC-13566), demonstrated an IC50 of 1.73 μM in PC3 cells transfected with WT-AR plasmid (Fig. 5a). Importantly, using a TR-FRET assay, we showed that VPC-13566 was able to displace a FITC labeled BAG1L peptide (residues 1-20) from the BF3 pocket of AR, proving its binding to the suggested site. However, the compound VPC-14449 [45], which targets the DNA binding domain of AR, was not able to displace BAG1L from its pocket (Fig. 5b). In the current study, we have employed the described luciferase-based assay to assess the transcriptional activity of 24 AR mutants in response to varying concentrations of VPC-13566 (Table 2, Additional file 4: Table S2). Under the same conditions, using an MTS cell proliferation assay, we assessed the toxicity of this compound in non-transfected PC3 cells and only noticed a mild toxicity at 50 μM (Fig. 5c). We hypothesized that the AR BF3 inhibitor should not be activated by any of the mutations, because they mainly cluster in the AR ABS, a region spatially distant from the BF3 site, and therefore, should not affect the interaction of VPC-13566 with the protein. Indeed, VPC-13566 effectively suppressed the transcriptional activity of all 24 AR mutants with the corresponding IC50 values in the range of 0.12 to 13.4 μM (Table 2, Additional file 4: Table S2).

## Discussion

### Repertoire and functionality of AR mutations

The AR is a multi-domain, ligand-inducible transcription factor composed of an N-terminal part, followed by a DNA binding (DBD) domain – the functional site of the receptor, a hinge region, and finally, a C-terminal LBD portion that is encoded by exons 5-8 and is known to be prone to mutations (Fig. 1) [46]. The incidence of AR mutations is rare in untreated prostate cancer, and is estimated to be in the range of 15 % in CRPC patients [47]. It has been reported that certain AR mutations can cause treatment failure of conventional AR antagonists, and can promote progression of PCa to its lethal CRPC state [13, 48, 49]. The accumulating evidence suggests that temporal monitoring of PCa patients being treated with AR pathway inhibitors can help detect the emergence of resistant AR mutants that drive CRPC.

Several of these CRPC-associated mutations are well documented in the literature, including hydroxyflutamide- and bicalutamide-resistant AR variants T878A and W742L/C, respectively. Other substitutions, including L702H [10], V716M [50], V731M [51], T878S [11], and H875Y [9], have been associated with receptor promiscuity – that is, an increased AR sensitivity to other steroids (progesterone, hydrocortisone, estradiol, and so on) or to AR antagonists. We have also recently reported four additional CRPC-associated variants, S889G, D880E, L882I, and E894K located on exon 8 of the AR gene [25].

In the current work, we employed a modified mutation-identification pipeline that allowed us to establish a detailed cfDNA-based mutation status of all 62 CRPC patients reported in [25], thus including 15 patients that were previously excluded from the analysis due to low DNA yield in their blood samples. The application of an improved sequence analysis approach allowed for the identification of four additional mutations in the AR (H875Q D891H, E898G, and T919S). We have also identified cases where two mutations occur on the same haplotype: T878A/D891H, T878A/S889G, F877L/T878A, H875Y/T878A, and H875Q/T919S. We hypothesized that understanding the potential clinical significance of these mutations would require *in vitro* functionalization to determine whether and how the mutations modulate the activity of the AR.

The measured responses of 24 single and double AR mutants to four AR antagonists, hydroxyflutamide, bicalutamide, enzalutamide, and ARN509, revealed that all these drugs can behave as AR agonists in the context of certain mutations (Table 3). In particular, the first generation antagonist hydroxyflutamide demonstrated an activating behavior towards the vast majority of the AR variants (with a notable exception of F877L), ranging from weak to strong agonist for mutations T878A/S, H875Y, F877L/T878A, T878A/D891H, and T878A/S889G.

Resistance to bicalutamide was also observed for the majority of the AR mutants, with the notable examples of W742L/C, T878A/S, S889G, D891H, and M896V/T mutations conferring strong bicalutamide activating phenotypes (Additional file 5: Figure S3). A partial agonist response to bicalutamide was observed for the majority of the studied mutants, especially when bicalutamide is present at high concentrations. In particular, when bicalutamide is administered above 16 μM, a profound activation was detected for single mutations V716M, V731M, H875Y, and for the mutation combinations T878A/D891H and T878A/S889G. Knowing that the steady state concentration of bicalutamide in the serum of prostate cancer patients is 8.9 μg/mL or approximately 20 μM [52], we were surprised to find that in our assay this drug activated the wild-type AR in the same concentrations range (approximately 16 μM) (Additional file 4: Table S2).

In addition to the documented case of enzalutamide-resistant mutation F877L, we identified that the combination F877L/T878A behaved as full agonist and did not show any inhibition in presence of enzalutamide. At higher doses of enzalutamide, we observed a stimulation of such mutants as T878A, H875Y, T878A/D891H, and T878A/S889G.

The functionalization of these 24 CRPC-associated AR mutants revealed that ARN509 generally exhibits dose-response profiles similar to those of enzalutamide, including agonist stimulation of mutants F877L, H875Y, and T878A.

These experiments indicate that any of the 24 AR mutants could drive resistance to at least one of the investigated drugs. These observations point to a complex and dynamic repertoire of AR mutations, driving therapeutic resistance in CRPC. This is underscored by the observation that the AR LBD haplotypes and haplotype ratios in cfDNA of individual CRPC patients can rapidly change in response to treatment regimens.

An interesting example is a patient VC-012 (Fig. 2). This individual underwent bicalutamide treatment that resulted in the development of CRPC. At this time-point, it was established that the patient’s AR harbored mutations M896V and S889G in the ratio of 47 % to 53 %, respectively. In our assay both of these mutations demonstrated profound resistance to bicalutamide (Fig. 2) and likely underlie the patient’s progression on bicalutamide. Importantly, both of these mutations were sensitive to enzalutamide and could be fully inhibited *in vitro* by 5 μM of the drug. Notably, patient VC-012 was switched to enzalutamide and after approximately 4 months of treatment, his cfDNA was collected and sequenced. At this time-point, the percentage of M896V and S889G had decreased to 4.7 % and 15.6 %, respectively. Remarkably, cfDNA sequencing revealed four new AR LBD mutations that emerged in response to enzalutamide administration. These new mutations included T878A (45.5 %), H875Y (7.4 %), and two double mutants F877L/T878A (21.4 %) and T878A/S889G (5.3 %). All four AR mutants demonstrated varying degrees of activation by enzalutamide in our assay (Fig. 2, Additional file 4: Table S2).

### Structural basis for agonistic conversion of bicalutamide and enzalutamide

The distinction between the agonistic and antagonistic actions of an AR ligand is conventionally attributed to the induced motion of helix 12 – one of the receptor’s folds forming the ABS cavity of the AR LBD. In a simplified view, AR inhibitors are believed to push helix 12 outward, preventing the formation of a ligand-locked, functional (agonist) configuration of the protein [53, 54]. Using methods of *in silico* modeling, we have investigated the possible structural basis for the agonist conversion of bicalutamide toward M896V and S889G mutants (Additional file 5: Figure S3), as well as the agonist interactions of T878A, H875Y, F877L/T878A, and T878A/S889G variants with enzalutamide (Fig. 4).

Our analysis of the distribution of the 15 mutated residues on the AR 3D structure demonstrated that they are mainly clustered around the AR steroid binding site and adjacent to helix 12 (Fig. 1). Previous structural studies into AR mutants have associated the T878A mutation with an increase in the size of the LBD cavity, the consequence of replacing a threonine residue with a more compact alanine residue. This size increase was attributed to the ability of the mutated AR to bind hydroxyflutamide in an agonist-like conformation [14]. Similarly, the F877L mutation was shown to increase the size of the AR ABS, thus enabling the receptor to accommodate the enzalutamide moiety [20].

Residue M896 belongs to the above-mentioned helix 12 of the AR, and is in close contact with the sulfonyl oxygen O15 of the bicalutamide molecule (1 Å), which makes the agonist conformation improbable with the WT-LBD (Additional file 3: Figure S2). The introduction of a less bulky non-polar valine residue into the 896-position should significantly increase the surface area of the ABS pocket, therefore allowing bicalutamide to bind in the closed agonist-like configuration. In a similar way, one could consider the effect of H875Y substitution: some preliminary modeling results showed that the hydroxyl group of the Y875 side chain can directly interact with -C(O)NHCH_3_ to further accommodate the ligand into the agonist configuration of the LBD.

The resistant character of the S889G substitution could be attributed to the increased mobility of helix 12 in the AR. In fact, the S889 residue is positioned right at the hinge of the helix 12, and introduction of the most flexible amino acid – glycine – into that position should significantly increase the mobility of the hinge, thus facilitating the motion of helix 12 toward the inbound ligand (Additional file 5: Figure S3).

We speculate that the double mutants F877L/T878A and T878A/S889G combine the agonist effects of the constituent single mutations. The intriguing possibility of synergism arising from the LBD compound mutations remains to be investigated.

### Targeting new sites on the AR could overcome the mutation-dependent drug resistance

The use of AR inhibitors that target the AR beyond its conventional and mutation-prone ABS could provide an effective strategy for addressing the problem of resistance, either alone or in combination with ABS-targeted agents such as enzalutamide. VPC-13566 has effectively inhibited all AR variants, including those that confer resistance to enzalutamide and to emerging drug candidates such as ARN509 (Table 2 and Additional file 4: Table S2). Thus, VPC-13566 could represent a viable treatment option for CRPC patients. Moreover, our AR BF3 inhibitor VPC-13566 exhibits a novel, distinctive mode of action against the AR, and therefore, could be considered for combinatorial therapy with conventional AR antagonists with a view to reducing toxicity and unfavorable side effects as well as delaying resistance of conventional single agent therapy.

## Conclusions

Genomic analysis of cfDNA is a minimally invasive method for interrogating mechanisms of therapeutic resistance in CRPC patients. In the present study, we demonstrate that cfDNA sequencing can identify mutations causally linked to resistance to therapies targeting the AR in CRPC. *In vitro* functionalization revealed that all 24 investigated AR mutations exhibited resistance to at least one of four AR inhibitors used in clinical practice. Moreover, some of the newly identified double AR mutants exhibited enhanced activation in presence of enzalutamide and ARN509 and/or demonstrated elevated sensitivity to stimulation by DHT or others steroids. These results underscore the importance of developing therapeutics that target the AR at sites outside the ABS. Thus, it is significant that the AR inhibitor VPC-13566, targeting the AR BF3 pocket, can effectively block the activity of all 24 AR mutants identified in CRPC patients. It will now be important to determine if co-targeting the AR with VPC-13566 and, for example, enzalutamide, can delay/overcome the resistance. Finally, the results of this study suggest that precision oncology for the improved management of CRPC patients may be a feasible option to improve patient care.

## Methods

### Patient cohort and sequencing of exon 8 of the AR gene

The cohort of 62 patients with metastatic CRPC recruited at the British Columbia Cancer Agency (BCCA) - Vancouver Prostate Centre (VPC, Vancouver, BC, Canada) between August 2013 and March 2014 was described previously [25]. In total, 19/62 (30 %) of patients were switched onto enzalutamide after collection of cfDNA. Clinico-pathological characteristics including prior and subsequent therapies were recorded for each patient. Blood collection, DNA isolation, and quantification were performed as described previously [25].

For this paper we have sequenced blood samples from 15 patients that were reported as not sequenced in the previous manuscript due to either low DNA yield (less than 30 ng as determined by Qubit 2.0 measurement using Qubit dsDNA HS Assay Kit) or repeatedly failed to produce useable sequencing data (VC-024, VC-028 and VC-044). DNA was amplified with the Sigma WGA2 kit (Cat No WGA2-10rxn or WGA2-50rxn) as per manufacturer’s instructions. The amplified material was sequenced with the Roche 454 GS FLX+ system, software version 2.9 as described in [25]. The amplicons from three WGA2 samples with detected mutations were also sequenced using an Illumina MiSeq sequencer; in all cases the results were concordant.

### Mutation calling

The biggest challenge in analysis of WGA2 amplified DNA is the significantly higher noise levels introduced by the genomic amplification. Therefore, we modified our pipeline to assure tighter control and more precise filtering of low-quality sequencing data. Raw sequence reads were mapped to the human genome (hg19) using BWA and visualized using the Integrative Genomics Viewer (IGV) [55]. All possible non-reference bases with greater than 25 Phred score at all amplicon locations were quantified using bam-readcount v. 0.7.4 (https://github.com/genome/bam-readcount) that allows for direct filtering of reads at each base position based on both sequence phred quality score (only bases with phred score >25 were scored) and mapping score (only bases with mapping score of >18 were scored). The raw base counts for each qualified base were converted to percentage relative to sequence coverage at corresponding position. We defined mutation candidates as non-reference bases with percentage value greater than 4 standard deviations distant to the mean and exceeding 0.1 % and 1 % level in non-amplified samples and amplified samples, respectively, due to increased background of base substitutions in WGA2 data. All calls detected in more than 75 % of sequenced samples were discarded as artifacts. We performed manual curation of all detected calls, in some cases adding calls that were present in other sequenced samples from the same patient, provided that they could be unambiguously identified as outliers on scatter plots of matching samples. Finally, we performed haplotype frequency estimation through manual curation of aligned reads in IGV. Reads with >7 mismatches with the reference sequence were considered uninformative for samples with multiple mutation calls.

### Constructs

Full-length human AR (WT-AR) was encoded on a pcDNA3.1 expression plasmid (Life technologies). The LBD point mutations (single and multiple) were generated using the QuikChange II Site-Directed Mutagenesis Kit (Agilent Technologies) as per manufacturer’s instructions using WT-AR as the template. The mutagenic oligonucleotide primers were designed individually with the desired mutation in the middle of the primer with approximately 10 to 15 bases of correct sequence on both sides.

### Steroid activation assay

PC3 cells lacking the AR and authenticated by IDEXX Laboratories (Maine, USA) were maintained in RPMI 1640 media (Life Technologies) and 5 % FBS (Hyclone Thermo Fisher Scientific) at 37 °C and 5 % CO2. Cultures were routinely monitored for mycoplasma contamination. For the steroid activation assay, cells were seeded in 96-well plates (5,000 cells/well) in RPMI 1640 medium with 5 % charcoal-stripped serum (CSS) (Hyclone). After 24 h, cells were co-transfected with 25 ng of wild-type or mutated AR and 25 ng of the reporter plasmid pARR3-tk-luciferase using TransIT20/20 transfection reagent (3 μL/μg of DNA) (Mirus Bio LLC, Madison, WI, USA) in Optimem serum-free media (Life Technologies) for 48 h according to manufacturer’s suggested protocol. Cells were stimulated with increasing concentrations of DHT, estradiol, progesterone, or hydrocortisone in 100 % ethanol (0 to 500 nM). Control cells were treated with 100 % ethanol alone. At 24 h after treatment, the medium was aspirated off and the cells were lysed by adding 60 μL of 1× passive lysis buffer (Promega) followed by shaking at room temperature for 15 min and two freeze/thaw cycles at -80 °C . Twenty microliters of lysate from each well were transferred onto a 96-well white flat bottom plate (Corning) and the luminescence signal was measured after adding 50 μL of luciferase assay reagent (Promega). The chemical oxidation of luciferin into oxyluciferin by the luciferase is accompanied by light production that can be quantified as luminescence by a TECAN M200Pro instrument. Each concentration was assayed in quadruplicate n = 4, with a biological replicate of n = 3. For each steroid, results were averaged and normalized by expressing them as a percentage of WT AR activity.

### AR inhibition assay

PC3 cells were seeded and transfected as described above. At 48 h after transfection, medium was aspirated and replaced with medium containing 0.1 nM R1881 and either 0.1 % DMSO (control) or serial dilutions of increasing concentrations of AR inhibitors ranging from 0 μM to 50 μM (hydroxyflutamide, bicalutamide, ARN509, enzalutamide, and VPC-13566). A non-stimulated/no R1881 control was used. After 24 h, cells were lysed and AR-dependent luciferase activity was quantified. Each concentration was assayed in quadruplicate n = 4, with a biological replicate of n = 3. Results were averaged and normalized by expressing them as a percentage of WT AR activity.

### Western blotting

Twenty microliters of each of the four replicates of DMSO/control-treated lysate from the luciferase assay were pooled with 20 uL of 5X sample buffer, boiled for 5 min, and 20 uL of the mixture was loaded on a 10 % SDS-PAGE gel and electrophoresed at 120 V for 90 min. Proteins were transferred to PVDF membrane (Millipore) at 100 V for 1.5 h at 4 °C, blocked for 1 h at room temperature with 5 % non-fat skim milk in TBS, followed by incubation with 1/1,000 dilution of AR N20 antibody (sc-816, Santa Cruz Biotechnologies) overnight at 4 °C. Membranes were incubated with 1/5,000 dilution of goat anti-rabbit IgG HRP (sc-2030, Santa Cruz Biotechnologies) for 1 h at room temperature, washed five times with TBS 0.1 % Tween 20 (Sigma), and bands visualized using Super Signal West Femto (Thermo Scientific) and a digital imager (Syngene G Box).

### Lanthascreen TR-FRET displacement assay

The displacement of a fluorescein isothiocyanate (FITC) labeled BAG1L peptide (FITC-MAQRGGARRPRGDRERLGSR) from the BF3 pocket of the LBD by the compounds VPC13566 and VPC14449 was assessed using a Time-Resolved Fluorescence Energy Transfer (TR-FRET). Compounds were tested in the range of 0.41 to 100 μM in a final concentration of 1.5 % DMSO. The protein AR-LBD, the FITC-BAG1L peptide, and the LanthaScreen® Elite Terbium-labeled anti-His-tag antibody (Life Technologies, PV5863) were used at final concentrations of 100 nM, 500 nM, and 5 nM, respectively. Briefly, the (His)6-tagged AR-LBD was prepared at 4X final concentration in the buffer (150 mM Li2SO4, 50 mM HEPES pH7.5, 10 % Glycerol, 20 μM of DHT and 0.5 mM tris(2-carboxyethyl)phosphine) in the presence of 4X Tb-anti-His-antibody (Mix A). Mix B contained 4X FITC-BAG1L peptide in 2 % DMSO. A three-fold serial dilution of the compounds was prepared at 100X final concentration in DMSO. The compounds were then diluted 50-fold in buffer to get a 2X final concentration and 2 % DMSO (Mix C). In a black flat bottom 384-well plate 5 μL of Mix A, 5 μL of Mix B, and 10 μL of Mix C were added.

The plate was incubated at room temperature for 2 h and FRET was analyzed on Synergy-4 multi-plate reader with the following settings: excitation, 340 nm; emission, 495 nm and 520 nm. The emission ratio (520:495) was analyzed, normalized to the buffer, and plotted.

### Cell viability assay

PC3 cells were plated at 5,000 cells per well in RPMI 1640 containing 5 % CSS in a 96-well plate and treated, after 72 h, with 0.1 nM R1881 and VPC-13566 (0-50 μM). After treatment for 24 h, cell density was measured using the 3-(4, 5-dimethylthiazol-2-yl)-5-(3-carboxymethoxyphenyl)-2-(4-sulfophenyl)-2H-tetrazolium assay according to the manufacturer’s protocol (CellTiter 961 Aqueous One Solution Reagent, Promega). The percentage of cell survival was normalized to the cell density of control wells treated by vehicle and 0.1 nM R1881.

### Availability of supporting data

All sequence data supporting the results of this article are available in the European Nucleotide Archive (ENA) repository, with the study accession number PRJEB12109 and a direct URL: http://www.ebi.ac.uk/ena/data/view/PRJEB12109.

### Ethics

This study was approved by the University of British Columbia Clinical Research Ethics Board (CREB) with the certificate approval number H09-01628 (renewed on 7 December 2015) and by the Vancouver Coastal Health Research Institute (VCHRI) with the certificate number V09-0320. All patients provided informed written consent. All the experimental methods comply with the Helsinki Declaration.

## Additional files

Additional file 1: Table S1. The results of the validation of a subset of detected mutations on MiSeq, Illumina. The run was designed to test 23 mutations in 11 cfDNA samples (both amplified and non-amplified). WGA samples are marked with *, n/a – sample not sequenced on MiSeq. Only two calls were not supported on MiSeq. S889G call in VC-012-t1 unamplified cfDNA was not detected on original 454 run, or on MiSeq resequencing. (DOCX 23 kb) Additional file 2: Figure S1. Western blot showing expression level of the CRPC-associated AR mutants in PC3 transfected cells. (TIF 351 kb) Additional file 3: Figure S2. Activation of wild-type AR by dihydrotestosterone (DHT), progesterone, estradiol, and hydrocortisone. The graphs represent the average ± SEM of three independent experiments with four replicates each. (TIF 252 kb) Additional file 4: Table S2. The response of the CRPC-associated mutants to increasing concentrations of anti-androgens. Abiraterone, bicalutamide, hydroxyflutamide, enzalutamide, ARN509, and an in-house developed AR inhibitor VPC-13566 were tested against the 24 CRPC-associated AR mutants. Each concentration was assayed in quadruplicate n = 4, with a biological replicate of n = 3. Results were averaged and normalized by expressing them as a percentage of WT AR activity ± SEM. (PDF 345 kb) Additional file 5: Figure S3. AR mutants associated with bicalutamide resistance in CRPC patients. (a) The AR LBD (cartoon representation, in gray) in complex with bicalutamide (ball-and-stick representation, in blue). The residues presented as gray sticks presented an agonist effect in the presence of bicalutamide in luciferase reporter transcription assay. The B ring of bicalutamide occupies the position that would normally be filled by the indole ring of tryptophan in the non-mutated W741 position (shown in transparent gray) in the LBD. (B) AR mutants showing agonist responses to bicalutamide by *in vitro* functional characterization. Each concentration was assayed in quadruplicate n = 4, with a biological replicate of n = 3. Results were averaged and normalized by expressing them as a percentage of WT AR activity ± SEM. (TIF 1129 kb)

## Abbreviations

ABS: Androgen binding site
AR: Androgen receptor
BF3: Binding function 3
cfDNA: Cell-free DNA
CRPC: Castration-resistant prostate cancer
DHT: Dihydrotestosterone
LBD: Ligand binding domain
PCa: Prostate cancer
