# Supplementary material for: Functional analysis of androgen receptor mutations that confer anti-androgen resistance identified in circulating cell-free DNA from prostate cancer patients
Source: Genome Biol. 2016 Jan 26;17:10. doi: 10.1186/s13059-015-0864-1 (PMC4729137; doi:10.1186/s13059-015-0864-1)
Supplement: Additional file 1: Table S1. — The results of the validation of a subset of detected mutations on MiSeq, Illumina. The run was designed to test 23 mutations in 11 cfDNA samples (both amplified and non-amplified). WGA samples are marked with *, n/a – sample not sequenced on MiSeq. Only two calls were not supported on MiSeq. S889G call in VC-012-t1 unamplified cfDNA was not detected on original 454 run, or on MiSeq resequencing. (DOCX 23 kb) [file 13059_2015_864_MOESM1_ESM.docx]

# Supplementary information

**Validation of mutation calling pipeline**

The modified mutation calling pipeline was first validated on the same data set as in [[25](#_ENREF_25)]. We have not observed any mutation candidates in normal genomic DNA samples (isolated from buffy coat) and obtained expected results in the three control cell lines: no mutations in AR exon 8 in LAPC4 known to have a wild-type AR gene, H875Y mutation at essentially 100 % frequency in 22RV1 and T878A mutation in LNCaP.

Next we have sequenced matched non-WGA and WGA samples from five patients (VC-012, VC-016, VC-017, VC-040, and VC-045). We detected H875Y mutation in both non-WGA and WGA samples in VC-040, and M896V in both samples of the first time point in VC-012 (we detected an additional S889G mutation in VC-012-t1 WGA sample). However, due to generally higher noise levels in WGA samples, we were unable to detect T878A mutation in VC-017 WGA. We did not detect mutations in both WGA and non-WGA VC-016 and VC-045 DNA samples. The analysis of the noise levels in matched WGA/non-WGA DNA samples showed that in WGA samples it was 10-15 times higher, therefore we set the cutoff value for calling mutations in WGA samples to at least 1 % non-reference reads reported by bam-readcount.

Next, we were able to obtain a second blood sample for three of our patients (VC-001, VC-012, and VC-041) 2 to 3 months after initial sampling, when they were switched to alternative anti-androgen protocol. We detected complete concordance in patients VC-001 and VC-041 (L874L, H875Y, and T878A in VC-001; H875Y in VC-041). However, we detected three additional mutations in VC-012 (originally M896V was identified in both t1 time point samples, S889G in WGA t1 sample, and we identified H875Y, F877L, T878A, S889G, and M896V in t2 time point).

Finally, in order to estimate robustness of the whole 454 sequencing and mutation detection pipeline, we have sequenced a subset of WGA-amplified and non-amplified samples on Illumina MiSeq. Unfortunately, due to scarcity of the original cfDNA we were not able to perform *de novo* WGA amplification. The results of the validation run are presented in Supplementary Table 1 (including results reported in [[25](#_ENREF_25)]). Only two mutation calls out of total 23 tested were not supported by the MiSeq results: D880E in VC-022 (the original estimated frequency 0.11 %) and T878A in VC-017 (originally called at 0.78 %) most probably due to somewhat higher noise level for the MiSeq run. All other calls were unambiguously confirmed, including three WGA2 samples with detected mutations reported in this manuscript.

| **Sample** | **Base change** | **Amino acid change** | **Mutant read count** | **Wild-type read count** | **Total read count** | **Percent mutant** | **Detected in MiSeq WGA2** | **Detected in MiSeq cfDNA** |
| --- | --- | --- | --- | --- | --- | --- | --- | --- |
| VC-001-t1* | ChrX:66943542 G>C | Silent | 368 | 13,021 | 13,396 | 2.75 | **Yes** | n/a |
|  | ChrX:66943543 C>T | H875Y | 287 | 13,100 | 13,387 | 2.14 | **Yes** | n/a |
|  | ChrX:66943552 A>G | T878A | 290 | 12,980 | 13,270 | 2.19 | **Yes** | n/a |
| VC-001-t2 | ChrX:66943542 G>C | Silent | 507 | 12,828 | 13,338 | 3.80 | n/a | **Yes** |
|  | ChrX:66943543 C>T | H875Y | 230 | 13,077 | 13,307 | 1.73 | n/a | **Yes** |
|  | ChrX:66943552 A>G | T878A | 418 | 12,238 | 12,656 | 3.30 | n/a | **Yes** |
|  | ChrX:66943591 G>C | D891H | 166 | 10,375 | 10,541 | 1.57 | n/a | **Yes** |
| VC-005 | ChrX:66943600 G>A | E894K | 170 | 10,745 | 10,915 | 1.40 | n/a | **Yes** |
| VC-012-t1* | ChrX:66943585 A>G | S889G | 307 | 4,769 | 5,076 | 6.05 | **Yes** | ***No*** |
|  | ChrX:66943606 A>G | M896V | 273 | 5,838 | 6,111 | 4.47 | **Yes** | **Yes** |
| VC-012-t1 | ChrX:66943606 A>G | M896V | 1,270 | 5,985 | 7,255 | 17.07 | n/a | **Yes** |
| VC-012-t2 | ChrX:66943543 C>T | H875Y | 49 | 10,355 | 10,404 | 0.47 | n/a | **Yes** |
|  | ChrX:66943549 T>C | F877L | 141 | 10,247 | 10,388 | 1.41 | **Yes** | **Yes** |
|  | ChrX:66943552 A>G | T878A | 476 | 9,584 | 10,060 | 4.97 | **Yes** | **Yes** |
|  | ChrX:66943585 A>G | S889G | 138 | 8,202 | 8,305 | 1.70 | **Yes** | **Yes** |
|  | ChrX:66943606 A>G | M896V | 31 | 8,934 | 8,965 | 0.41 | n/a | **Yes** |
| VC-017 | ChrX:66943552 A>G | T878A | 99 | 12,626 | 12,725 | 0.78 | n/a | ***No*** |
| VC-021* | ChrX:66943545 T>G | H875Q | 251 | 9,846 | 10,097 | 2.49 | **Yes** | n/a |
|  | ChrX:66943675 A>T | T919S | 238 | 9,004 | 9,242 | 2.58 | **Yes** | n/a |
| VC-022 | ChrX:66943560 C>A | D880E | 12 | 10,902 | 10,914 | 0.11 | n/a | ***No*** |
| VC-041-t2 | ChrX:66943543 C>T | H875Y | 4,665 | 15,662 | 20,327 | 23.22 | **Yes** | n/a |
| VC-063 | ChrX:66943543 C>T | H875Y | 270 | 14,874 | 15,144 | 1.84 | n/a | **Yes** |
|  |  |  |  |  |  |  |  |  |
| * - WGA2 sample | |  |  |  |  |  |  |  |
| n/a - sequencing not run | |  |  |  |  |  |  |  |

**Supplementary Table 1:** **The results of the validation of a subset of detected mutations on MiSeq, Illumina.** The run was designed to test 23 mutations in 11 cfDNA samples (both amplified and non-amplified). WGA samples are marked with *, n/a – sample not sequenced on MiSeq. Only two calls were not supported on MiSeq. S889G call in VC-012-t1 unamplified cfDNA was not detected on original 454 run, or on MiSeq resequencing.
