# Supplementary figures and images for: Functional analysis of androgen receptor mutations that confer anti-androgen resistance identified in circulating cell-free DNA from prostate cancer patients
Source: Genome Biol. 2016 Jan 26;17:10. doi: 10.1186/s13059-015-0864-1 (PMC4729137; doi:10.1186/s13059-015-0864-1)

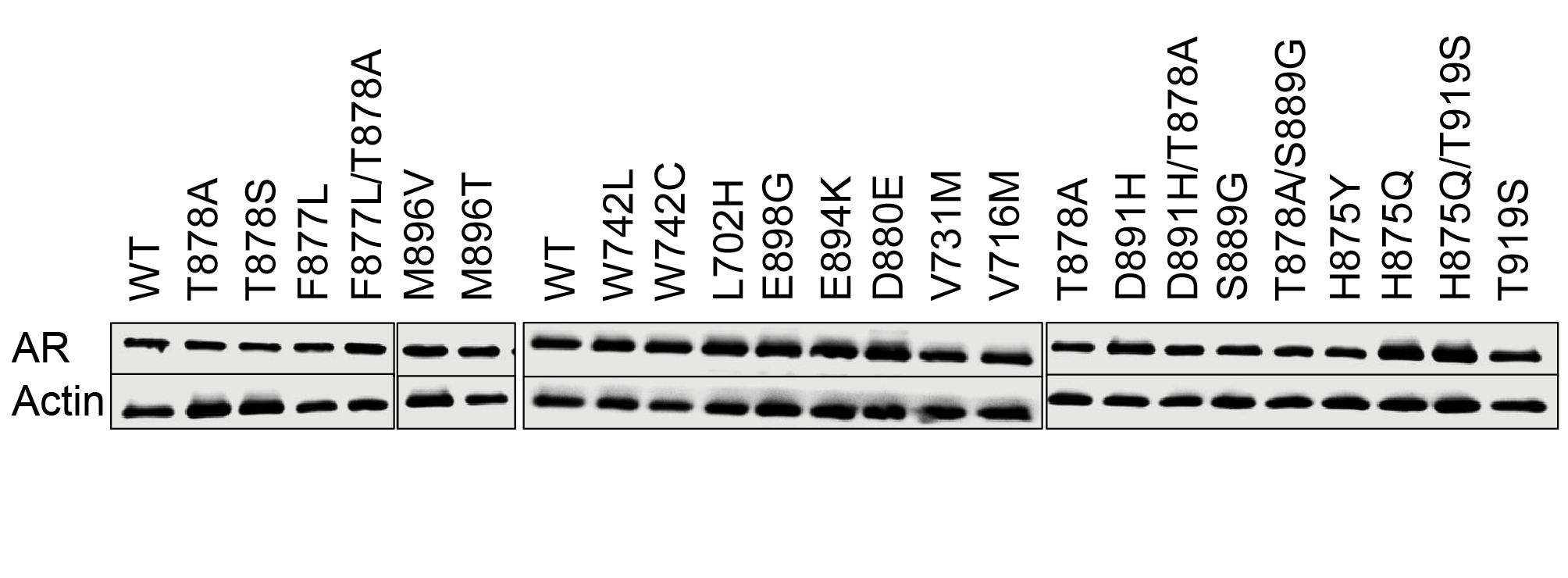

Supplement: Additional file 2: Figure S1. — Western blot showing expression level of the CRPC-associated AR mutants in PC3 transfected cells. (TIF 351 kb) [file 13059_2015_864_MOESM2_ESM.tif]

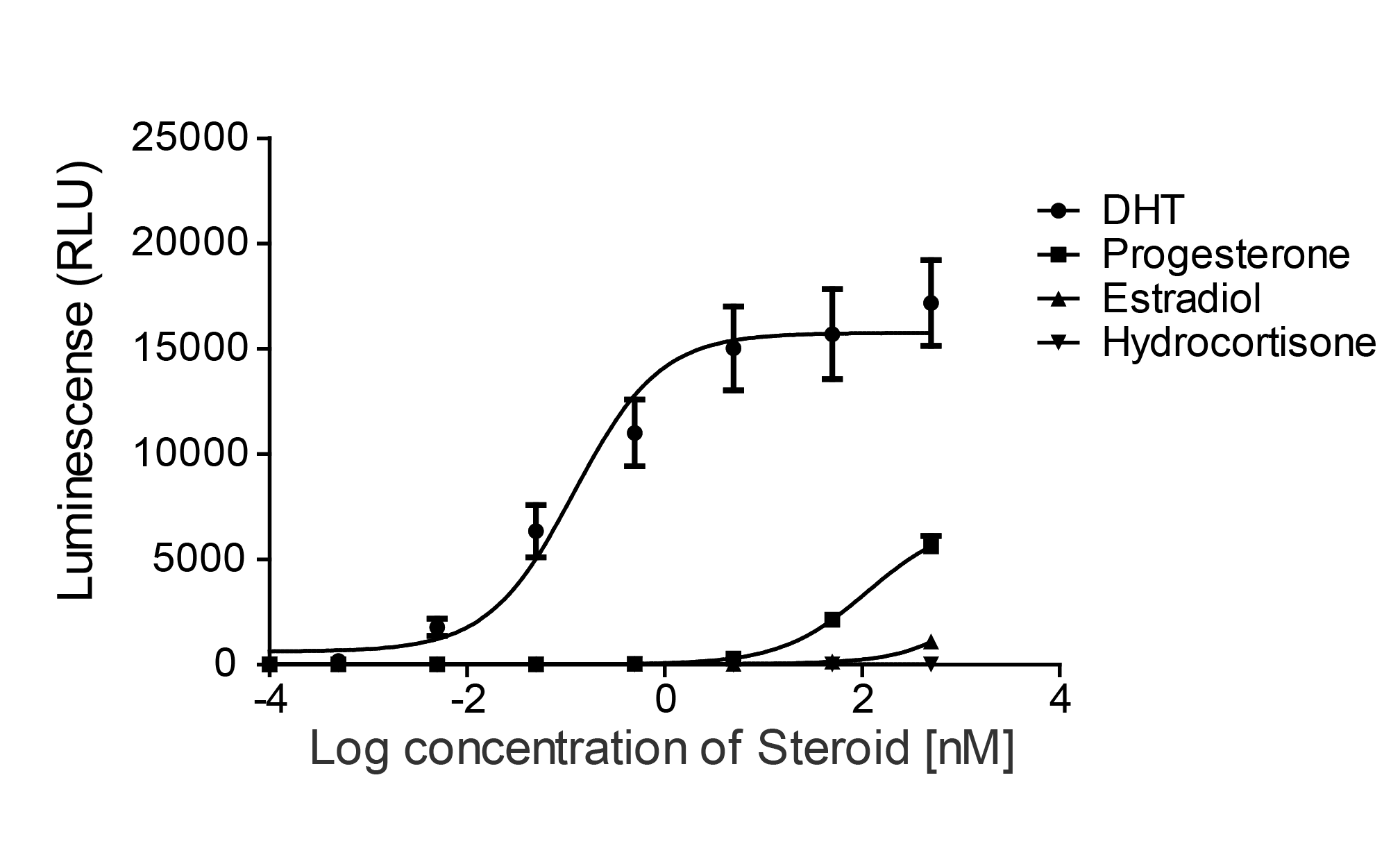

Supplement: Additional file 3: Figure S2. — Activation of wild-type AR by dihydrotestosterone (DHT), progesterone, estradiol, and hydrocortisone. The graphs represent the average ± SEM of three independent experiments with four replicates each. (TIF 252 kb) [file 13059_2015_864_MOESM3_ESM.tif]

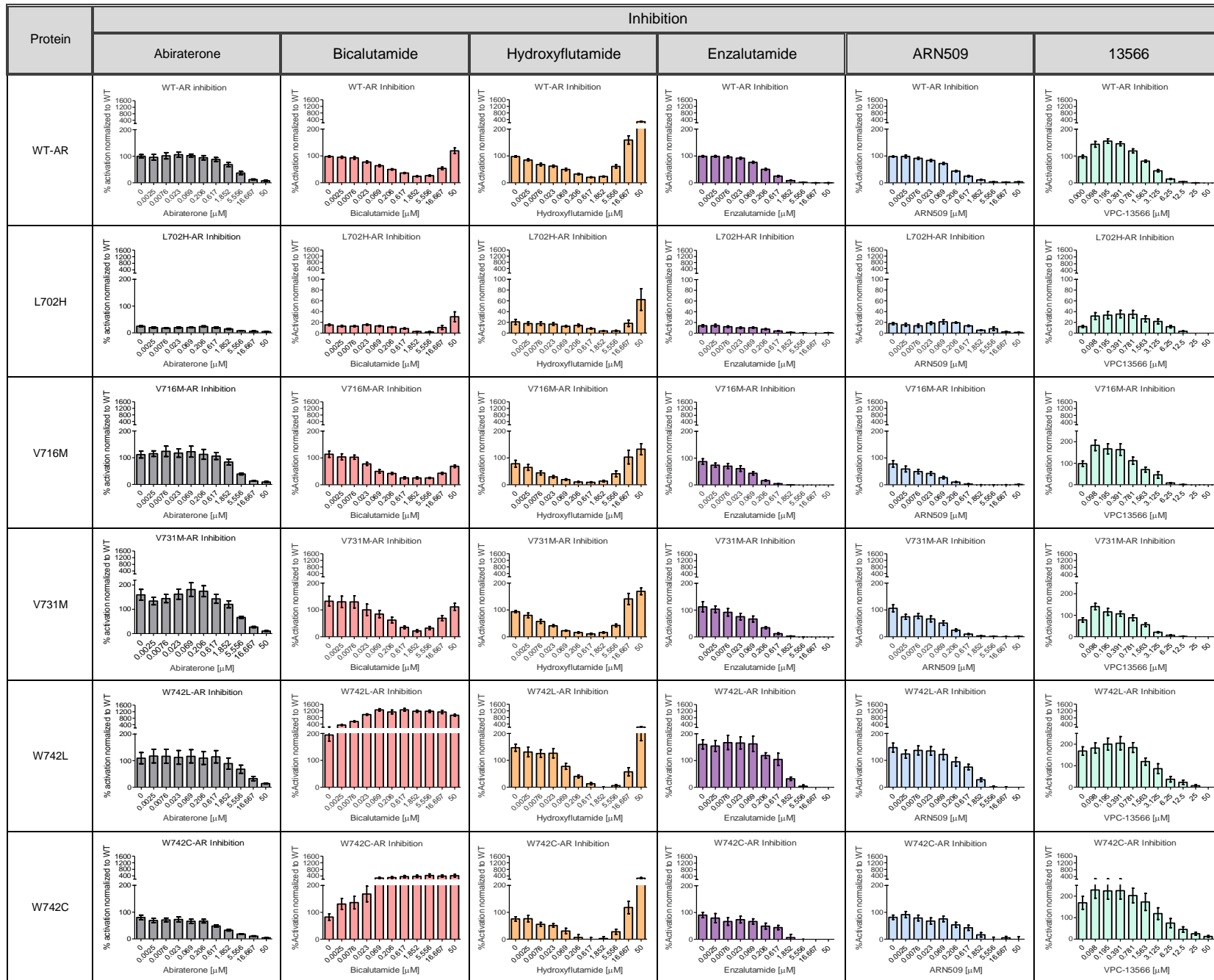

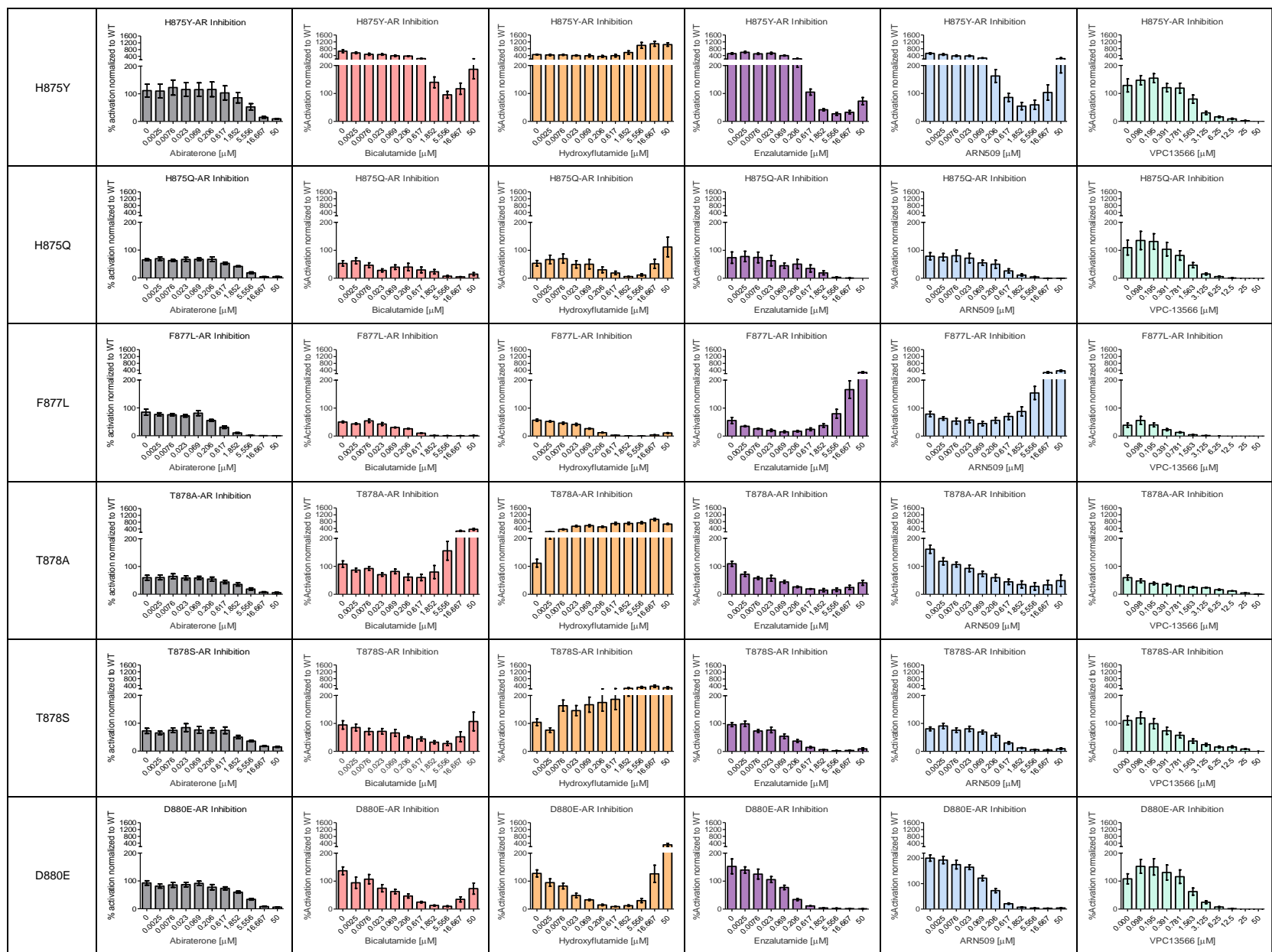



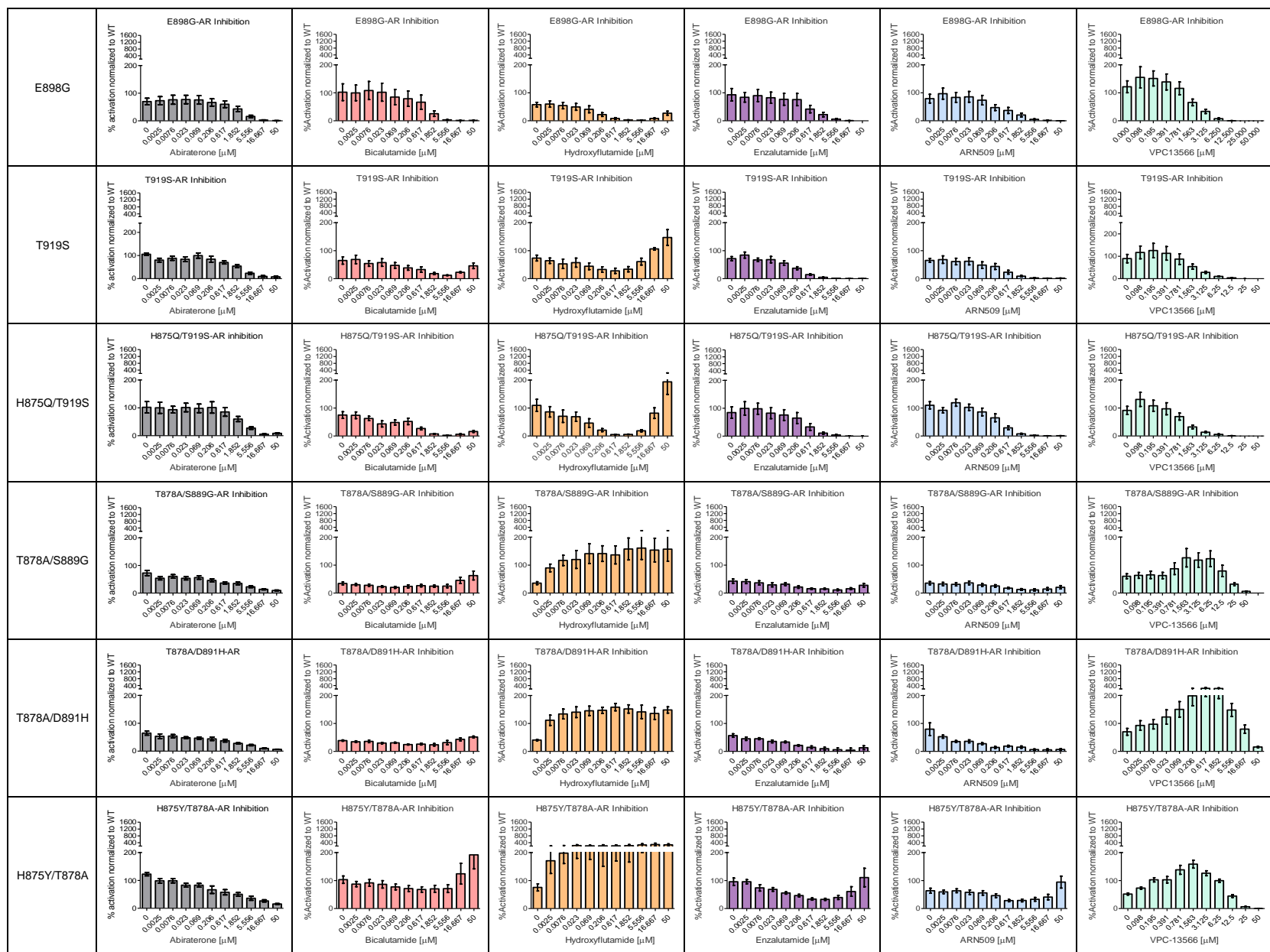

F877L/T878A

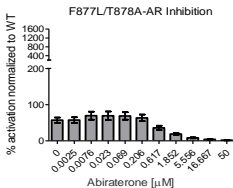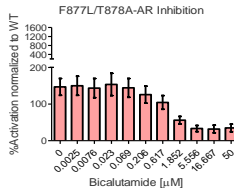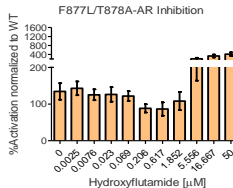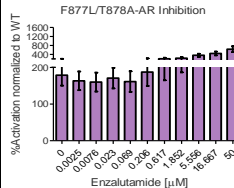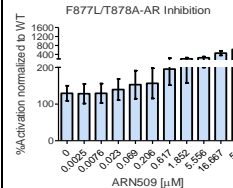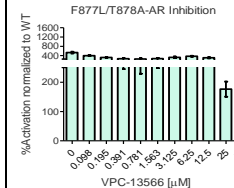

Supplement: Additional file 4: Table S2. — The response of the CRPC-associated mutants to increasing concentrations of anti-androgens. Abiraterone, bicalutamide, hydroxyflutamide, enzalutamide, ARN509, and an in-house developed AR inhibitor VPC-13566 were tested against the 24 CRPC-associated AR mutants. Each concentration was assayed in quadruplicate n = 4, with a biological replicate of n = 3. Results were averaged and normalized by expressing them as a percentage of WT AR activity ± SEM. (PDF 345 kb) [file 13059_2015_864_MOESM4_ESM.pdf]

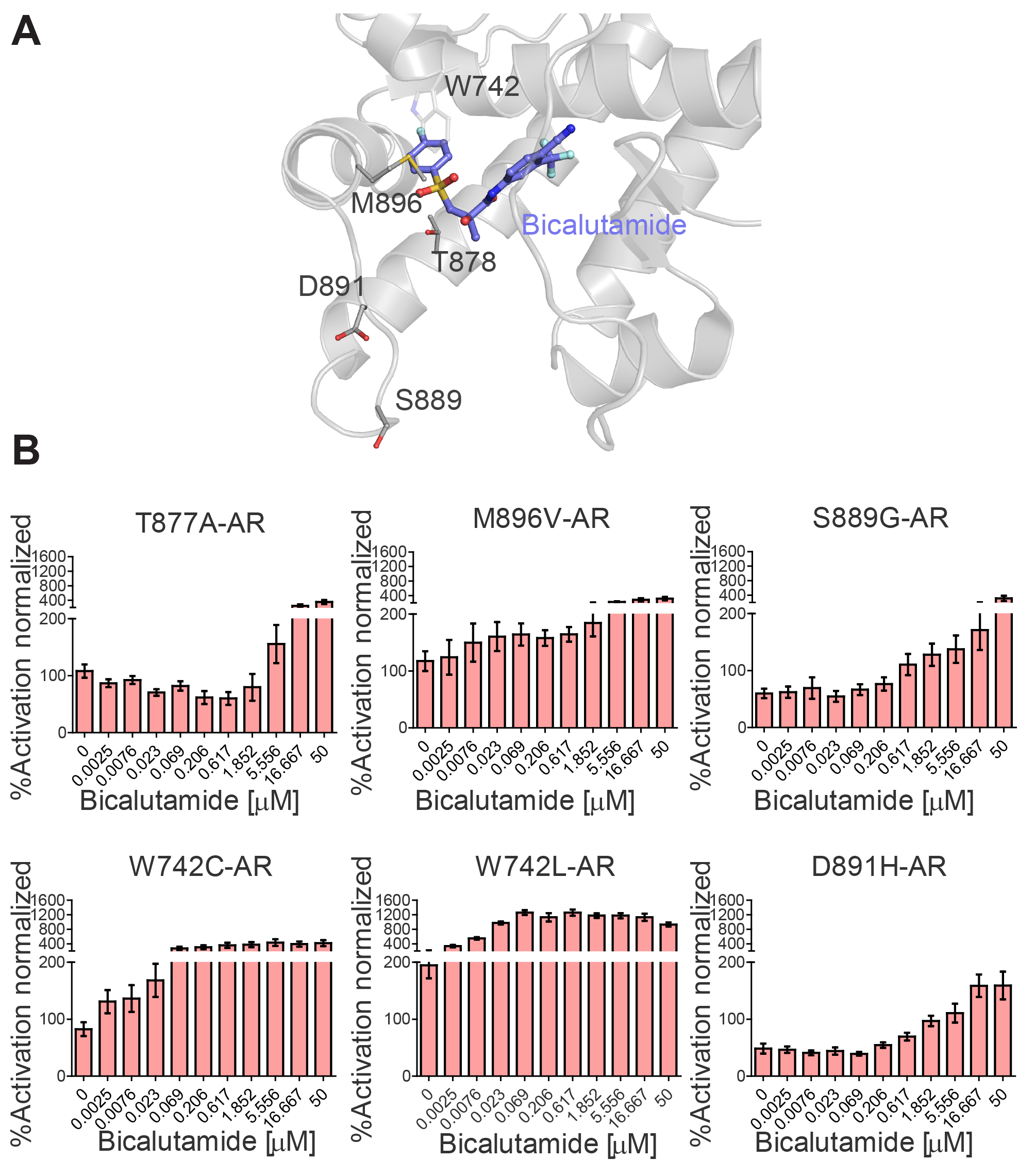

Supplement: Additional file 5: Figure S3. — AR mutants associated with bicalutamide resistance in CRPC patients. (a) The AR LBD (cartoon representation, in gray) in complex with bicalutamide (ball-and-stick representation, in blue). The residues presented as gray sticks presented an agonist effect in the presence of bicalutamide in luciferase reporter transcription assay. The B ring of bicalutamide occupies the position that would normally be filled by the indole ring of tryptophan in the non-mutated W741 position (shown in transparent gray) in the LBD. (B) AR mutants showing agonist responses to bicalutamide by in vitro functional characterization. Each concentration was assayed in quadruplicate n = 4, with a biological replicate of n = 3. Results were averaged and normalized by expressing them as a percentage of WT AR activity ± SEM. (TIF 1129 kb) [file 13059_2015_864_MOESM5_ESM.tif]
